# On Positive Solutions for the Rational Difference Equation Systems *x*_*n*+1_ = *A*/*x*_*n*_ *y*_*n*_^2^, and *y*_*n*+1_ = *By*_*n*_/*x*_*n*−1_ *y*_*n*−1_

**DOI:** 10.1155/2014/857480

**Published:** 2014-10-28

**Authors:** Hui-li Ma, Hui Feng

**Affiliations:** Department of Mathematics, Northwest Normal University, Lanzhou, Gansu 730070, China

## Abstract

Our aim in this paper is to investigate the behavior of positive solutions for the following systems of rational difference equations: *x*_*n*+1_ = *A*/*x*_*n*_ *y*_*n*_^2^, and *y*_*n*+1_ = *By*_*n*_/*x*_*n*−1_ *y*_*n*−1_, *n* = 0,1,…, where *x*_−1_, *x*_0_, *y*_−1_, and *y*_0_ are positive real numbers and *A* and *B* are positive constants.

## 1. Introduction

In recent years, with the wide application of computers, difference system has become one of the important theoretical bases for computer, information system, engineering control, ecological balance, and so forth. As typical nonlinear difference equations, rational difference equations have become a research hot spot in mathematical modelling. The behavior of solutions of the system for rational difference equation has received extensive attention.

In [1], Ozban has investigated the periodicity of solutions of the system of difference equations:

(1) $$\begin{array}{c} x_{n+1}=\frac{1}{y_{n-k}},\;y_{n+1}=\frac{y_{n}}{x_{n-m}y_{n-m-k}},\;n=0,1,\ldots . \end{array}$$

In [2], Kurbanlı et al. studied the behavior of the positive solutions of the system of difference equations:

(2) $$\begin{array}{c} x_{n+1}=\frac{x_{n-1}}{y_{n}x_{n-1}+1},\;y_{n+1}=\frac{y_{n-1}}{x_{n}y_{n-1}+1},\;n=0,1,\ldots . \end{array}$$

The periodicity of the positive solutions of the rational difference system

(3) $$\begin{array}{c} x_{n+1}=\frac{1}{y_{n}},\;y_{n+1}=\frac{y_{n}}{x_{n-1}y_{n-1}},\;n=0,1,\ldots , \end{array}$$

has been studied by Çinar in [3].

In [4], Ozban studied the behavior of the positive solutions of the system of difference equations

(4) $$\begin{array}{c} x_{n}=\frac{a}{y_{n-3}},\;y_{n}=\frac{by_{n-3}}{x_{n-q}y_{n-q}},\;n=0,1,\ldots . \end{array}$$

For similar research on difference systems, we refer the reader to [5, 6] and the references therein.

In this paper, we investigate the behavior of positive solutions for the system of rational difference equations

(5) $$\begin{array}{c} x_{n+1}=\frac{A}{x_{n}y_{n}^{2}},\;y_{n+1}=\frac{By_{n}}{x_{n-1}y_{n-1}},\;n=0,1,\ldots , \end{array}$$

where *A*, *B* ∈ [0, +*∞*) and *x*_−1_, *x*_0_, *y*_−1_, and *y*_0_ ∈ [0, +*∞*).

Before stating our main results, we state some definitions used in this paper.

**Definition 1 .**

A pair of sequences of positive real numbers {(*x*_*n*_, *y*_*n*_)}_*n*=0_^*∞*^ that satisfies (5) is called a positive solution of (5).

**Definition 2 .**

A solution {(*x*_*n*_, *y*_*n*_)}_*n*=0_^*∞*^ of (5) is periodic, if there exists a positive integer *T* such that (*x*_*n*_, *y*_*n*_) = (*x*_*n*+*T*_, *y*_*n*+*T*_), *n* = 0,1,…, and *T* is called a period.

## 2. Main Results

First, we study the periodic nature of positive solution of system (5).

**Theorem 3 .**

Let *x*_−1_, *x*_0_, *y*_−1_, and *y*_0_ be positive real numbers and let {(*x*_*n*_, *y*_*n*_)}_*n*=0_^*∞*^ be a solution of system (5). Then for *A* = *B* ^2^, *A*, *B* ∈ [0, +*∞*), all solutions of system (5) are periodic with period 3.

**Proof**

For *A*, *B* > 0, it can be seen easily that all solutions of (5) are positive. Thus, by (5) we have the following equality:

(6) $$\begin{array}{c} x_{n+1}=\frac{A}{x_{n}y_{n}^{2}},\;\;y_{n+1}=\frac{By_{n}}{x_{n-1}y_{n-1}}. \end{array}$$

Repeating application of (5) yields

(7)  xn+2=Axn+1yn+12=xnxn−12yn−12B2, yn+2=Byn+1xnyn=B2xnxn−1yn−1.

Similarly,

(8)  xn+3=Axn+2yn+22=AB2xn=xn, yn+3=Byn+2xn+1yn+1=B2Ayn=yn.

The proof is complete.

**Theorem 4 .**

Suppose that *x*_−1_, *x*_0_, *y*_−1_, and *y*_0_ are positive real numbers. Let {(*x*_*n*_, *y*_*n*_)}_*n*=0_^*∞*^ be a solution of system (5) with *x*_−1_ = *c*, *x*_0_ = *a*, *y*_−1_ = *d*, and *y*_0_ = *b*. Then for *A* = *B* ^2^, where *A* and *B* are positive constants, all solutions of system (5) are

(9) x3n+1=Aab2,  y3n+1=Bbcd,x3n+2=ac2d2B2,  y3n+2=B2acd,x3n+3=a,  y3n+3=b,n=0,1,….

**Proof**

For *A*, *B* > 0, it can be seen easily that all solutions of (5) are positive.

For *n* = 0 we have

(10)  x1=Ax0y02=Aab2,  y1=By0x−1y−1=Bbcd, x2=Ax1y12=ac2d2B2,  y2=By1x0y0=B2acd, x3=Ax2y22=a,  y3=By2x1y1=b.

Now suppose that *n* ∈ **Z** ^+^ and that our assumption holds for *n* − 1. One will show that the result holds for *n*. From system (5), we obtain

(11)  x3n+1=Ax3n−1+3y3n−1+32=Aab2, y3n+1=By3n−1+3x3n−1+2y3n−1+2=Bbcd.

Then,

(12)  x3n+2=Ax3n+1y3n+12=ac2d2B2, y3n+2=By3n+1x3n−1+3y3n−1+3=B2acd.

In particular, from Theorem 3, we get

(13)  x3n+3=x0+3n+1=x0=a, y3n+3=y0+3n+1=y0=b.

Therefore, the proof is complete.

**Example 5 .**

**Figure 1:**
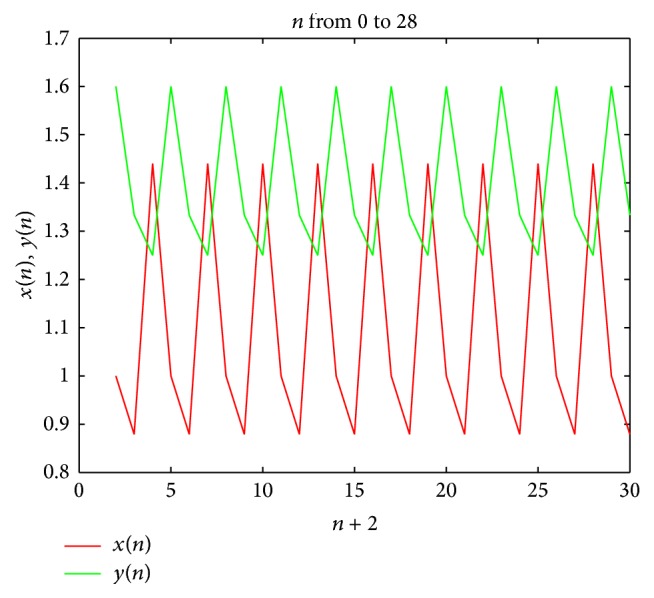


Set *A* = 2.25 and *B* = 1.5 and *x*_−1_ = 3, *x*_0_ = 1, *y*_−1_ = 0.6, and *y*_0_ = 1.6.  Figure 1 describes the periodic nature of system (5).

Next, we consider the case that *A* ≠ *B* ^2^, where *A* and *B* are positive constants.

**Theorem 6 .**

Let {(*x*_*n*_, *y*_*n*_)}_*n*=0_^*∞*^ be an arbitrary positive solution of (5).

(i) If *A* < *B* ^2^, then, for each integer *n* ≥ 0, the subsequence {*x*_*n*+3*l*_}_*l*=0_^*∞*^ → 0, and the subsequence {*y*_*n*+3*l*_}_*l*=0_^*∞*^ → *∞*.
(ii) If *A* > *B* ^2^, then, for each integer *n* ≥ 0, the subsequence {*x*_*n*+3*l*_}_*l*=0_^*∞*^ → *∞*, and the subsequence {*y*_*n*+3*l*_}_*l*=0_^*∞*^ → 0.
(iii) *x*_*n*+3_ *y*_*n*+3_ = *x*_*n*_ *y*_*n*_, *n* = 0,1,….

**Proof**

(i) For every fixed *n*, we will show that

(14) $$\begin{array}{c} x_{n+3l}={\left(\frac{A}{B^{2}}\right)}^{l}x_{n},\;l=0,1,2,\ldots . \end{array}$$

In fact, for *l* = 0, *x*_*n*_ = (*A*/*B* ^2^)^0^ *x*_*n*_. Assume that (14) holds for *l* − 1; that is,

(15) $$\begin{array}{c} x_{n+3l-3}={\left(\frac{A}{B^{2}}\right)}^{l-1}x_{n},\;l=0,1,2,\ldots . \end{array}$$

For *l*, we have the following:

(16) xn+3l=xn+3+3l−3=AB2l−1xn+3=AB2l−1AB2xn=AB2lxn.

Similarly, it can be obtained by induction that

(17) $$\begin{array}{c} y_{n+3l}={\left(\frac{B^{2}}{A}\right)}^{l}y_{n},\;l=0,1,2,\ldots . \end{array}$$

If *A* < *B* ^2^, we can get by (14) and (17) that

(18) $$\begin{array}{c} {\left\{x_{n+3l}\right\}}_{l=0}^{\infty }⟶0,\;\;{\left\{y_{n+3l}\right\}}_{l=0}^{\infty }⟶\infty . \end{array}$$

(ii) If *A* > *B* ^2^, by (14) and (17),

(19) $$\begin{array}{c} {\left\{x_{n+3l}\right\}}_{l=0}^{\infty }⟶\infty ,\;\;{\left\{y_{n+3l}\right\}}_{l=0}^{\infty }⟶0. \end{array}$$

(iii) From the proof of Theorem 3, we have

(20)  xn+3=Axn+2yn+22=AB2xn, yn+3=Byn+2xn+1yn+1=B2Ayn.

Multiplying both sides, respectively, yields *x*_*n*+3_ *y*_*n*+3_ = *x*_*n*_ *y*_*n*_, *n* = 0,1,….

**Example 7 .**

Let *x*_−1_ = 8, *x*_0_ = 1, *y*_−1_ = 3, and *y*_0_ = 7.

**Figure 2:**
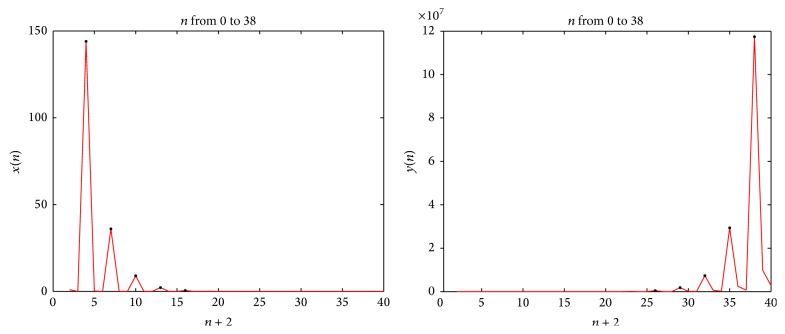


**Figure 3:**
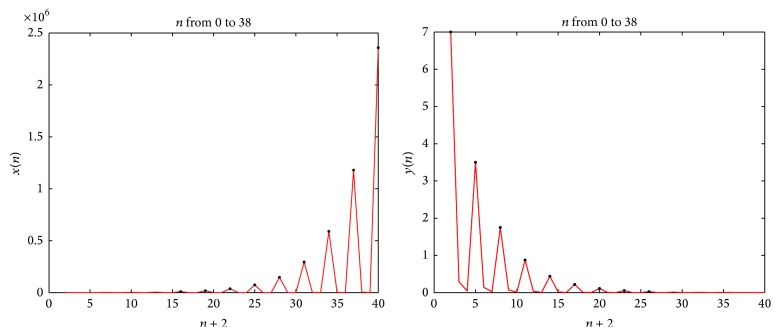


(i) For *A* = 1, *B* = 2, and *n* = 2, Figure 2 and Table 1 describe the behavior of the sequence {*x*_*n*+3*l*_}_*l*=0_^*∞*^ and {*y*_*n*+3*l*_}_*l*=0_^*∞*^ (shown by the black spot at the top of every peak).
(ii) For *A* = 2, *B* = 1, and *n* = 2, Figure 3 and Table 2 describe the behavior of the sequence {*x*_*n*+3*l*_}_*l*=0_^*∞*^ and {*y*_*n*+3*l*_}_*l*=0_^*∞*^ (shown by the black spot at the top of every peak).

## Figures and Tables

**Table 1:** 

*l*	0	1	2	3	4	5	6	7	*⋯*
*x*_2+3*l*_	144.00	36.00	9.00	2.25	0.56	1.41 × 10^−1^	3.52 × 10^−2^	8.79 × 10^−3^	*⋯*
*y*_2+3*l*_	0.17	0.67	2.67	10.67	42.67	1.71 × 10^2^	6.83 × 10^2^	2.73 × 10^3^	*⋯*

**Table 2:** 

*l*	0	1	2	3	4	5	6	7	*⋯*
*x*_2+3*l*_	5.76 × 10^2^	1.15 × 10^3^	2.30 × 10^3^	4.61 × 10^3^	9.22 × 10^3^	1.84 × 10^4^	3.69 × 10^4^	7.37 × 10^4^	*⋯*
*y*_2+3*l*_	4.17 × 10^−2^	2.08 × 10^−2^	1.04 × 10^−2^	5.21 × 10^−3^	2.60 × 10^−3^	1.30 × 10^−3^	6.51 × 10^−4^	3.26 × 10^−4^	*⋯*
